# Rare aneurysm of anterior mitral valve leaflet-a case report

**DOI:** 10.1186/s13019-019-1032-6

**Published:** 2019-11-27

**Authors:** Muhammad Tariq, Ibrahim Zahid, Shahid Sami

**Affiliations:** 1 Fellow Advance Complex Cardiac Surgery, St. Boniface Hospital, Winnipeg, Manitoba Canada; 2 Dow Medical College, Dow University of Health Sciences, Karachi, Pakistan; 3 Consultant Cardiothoracic Surgeon, Aga Khan University Hospital, Karachi, Pakistan

**Keywords:** Mitral valve aneurysm, Anterior mitral leaflet, Mitral regurgitation, Mitral valve replacement

## Abstract

**Background:**

Mitral valve aneurysm (MVA) is a saccular outpouching of the mitral leaflet which expands on systole and collapses during diastole. The case of MVA was first described in 1729 by Morand. It is one of the rare entities with a reported incidence of only 0.2–0.29% and no such case reported in Pakistan before.

**Case presentation:**

A 51 year old female presented with dyspnea and chest pain for 3 months. Upon investigating, trans-esophageal echocardiography (TEE) revealed thickened anterior mitral valve leaflet with rolled up margins, forming an aneurysm, with severe mitral regurgitation. Subsequently, the valve was evaluated intra-operatively for repair but eventually had to be excised and then successfully replaced with a bioprosthetic valve.

**Conclusions:**

TEE is an excellent technique to confirm the diagnosis of a mitral valve leaflet aneurysm, and depending upon the severity of the defect, valve repair can be attempted but replacement becomes the most suitable treatment modality, once repair is deemed impossible. We hereby report a rare case, where timely diagnosis, appropriate surgical intervention and regular post-operative follow up helped in achieving good prognosis of this rare entity.

## Background

Mitral valve aneurysm (MVA) is a saccular, bulging structure of the mitral leaflet which expands on systole and collapses during diastole [1]. The case of MVA was first described in 1729 by Morand [2]. Rupture of an aneurysm may result due to perforation in a longstanding MVA, leading to a communication with the left atrium, bringing about mitral regurgitation and heart failure.

We report a rare case of un-ruptured MVA causing mitral regurgitation in a middle aged female.

## Case presentation

A 51 year old female was admitted to our hospital with complaints of **dyspnea** on exertion and **chest pain** radiating towards the back and right arm for 3 months. She was a known case of hypertension and osteoarthritis of both knees, and had a positive family history for ischemic heart disease. On examination, the first and second heart sounds were normal and a **III/VI systolic murmur** was heard at the apex with radiation to the left axilla.

**Fig. 1:**
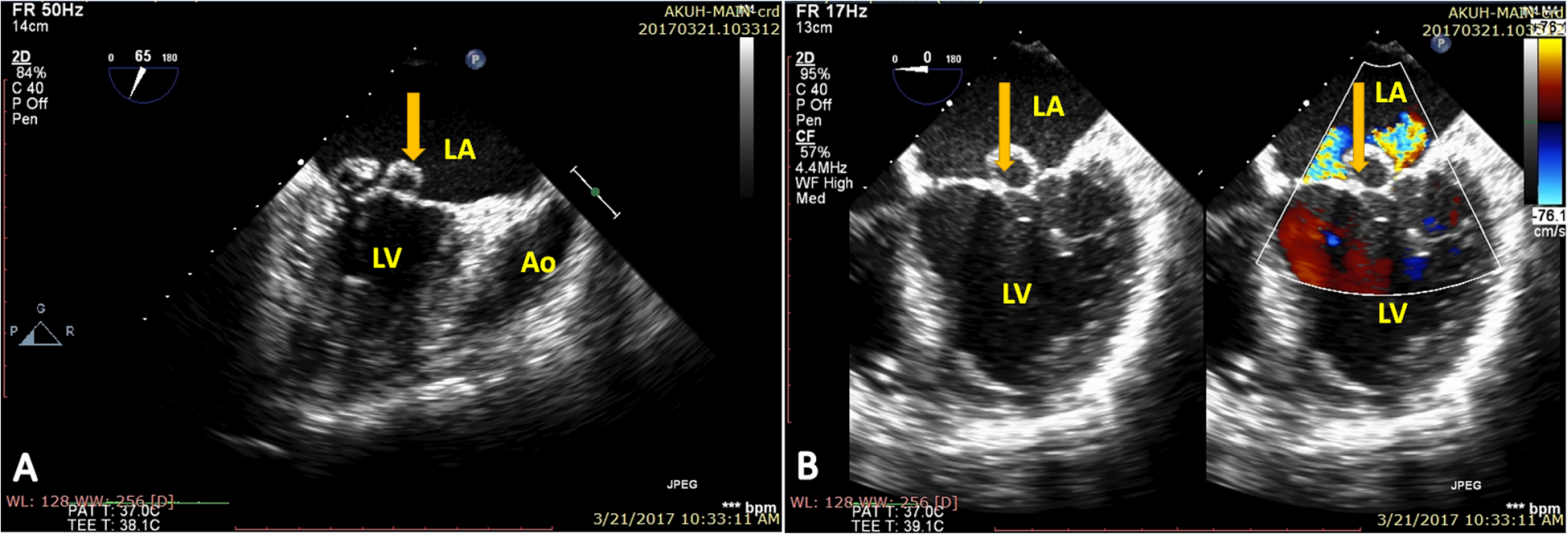
**a** A trans esophageal two chamber 2D echocardiogram revealing a saccular structure (yellow arrow) attached to the anterior mitral leaflet, consistent with mitral valve aneurysm. **b** A 2D trans esophageal echocardiogram (left) and color flow doppler (right) showing mitral valve leaflet aneurysm (yellow arrow) causing severe regurgitation. LA: Left Atrium, LV: Left Ventricle, Ao: Aorta

**Fig. 2:**
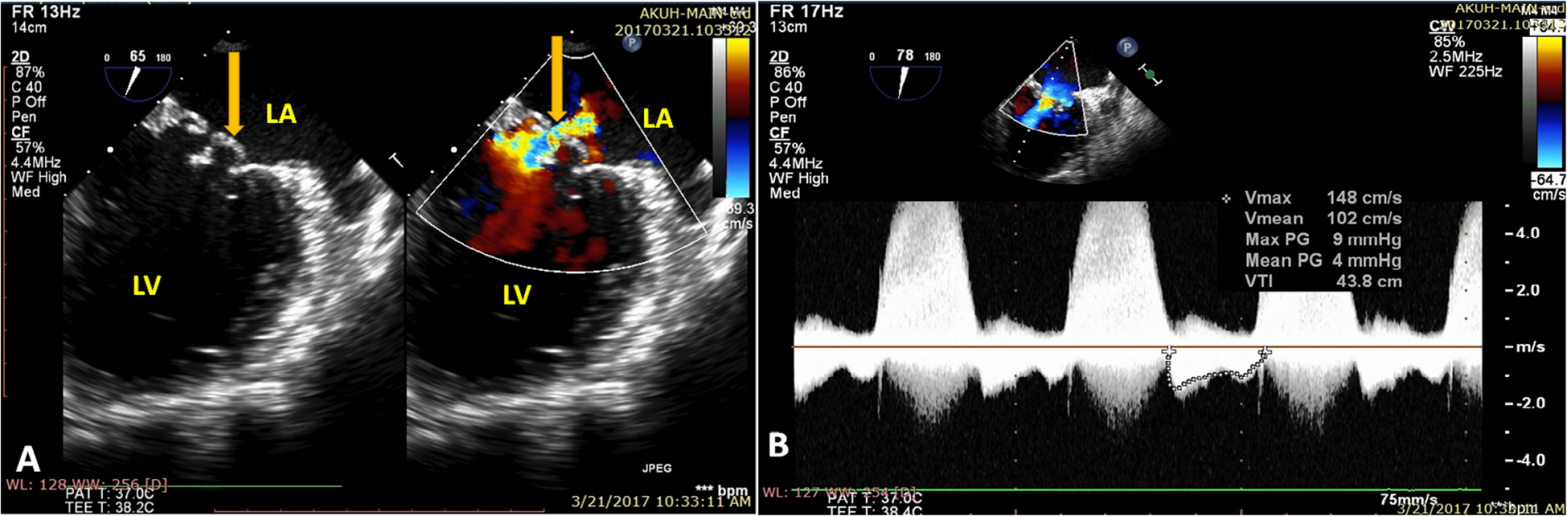
**a** The color Doppler flow showing that the aneurysm was filled with flow during systole (yellow arrow) and the color flow in the cystic mass was communicated with the left atrium. **b** Continuous wave Doppler revealing the aneurysm causing hemodynamic disturbance and an increase of mean pressure gradient. LA: Left Atrium, LV: Left Ventricle

Upon investigating, a transthoracic echocardiogram (TTE) showed thickened rolled up and prolapsed anterior mitral leaflet with severe mitral regurgitation (MR). A Trans-Esophageal Echocardiography (TEE) revealed thickened anterior mitral valve leaflet with rolled up margins, forming an aneurysm, with severe mitral regurgitation but no rupture of sub-valvular apparatus (Figs. 1 and 2). The posterior leaflet was also thickened with rolled up tips with mild prolapse of P1/P3. Mitral valve annulus measured about 42 mm. A diagnosis of mitral valve aneurysm along with severe MR was made, with prolapse of both leaflets and a grade II left ventricular diastolic dysfunction.

**Fig. 3:**
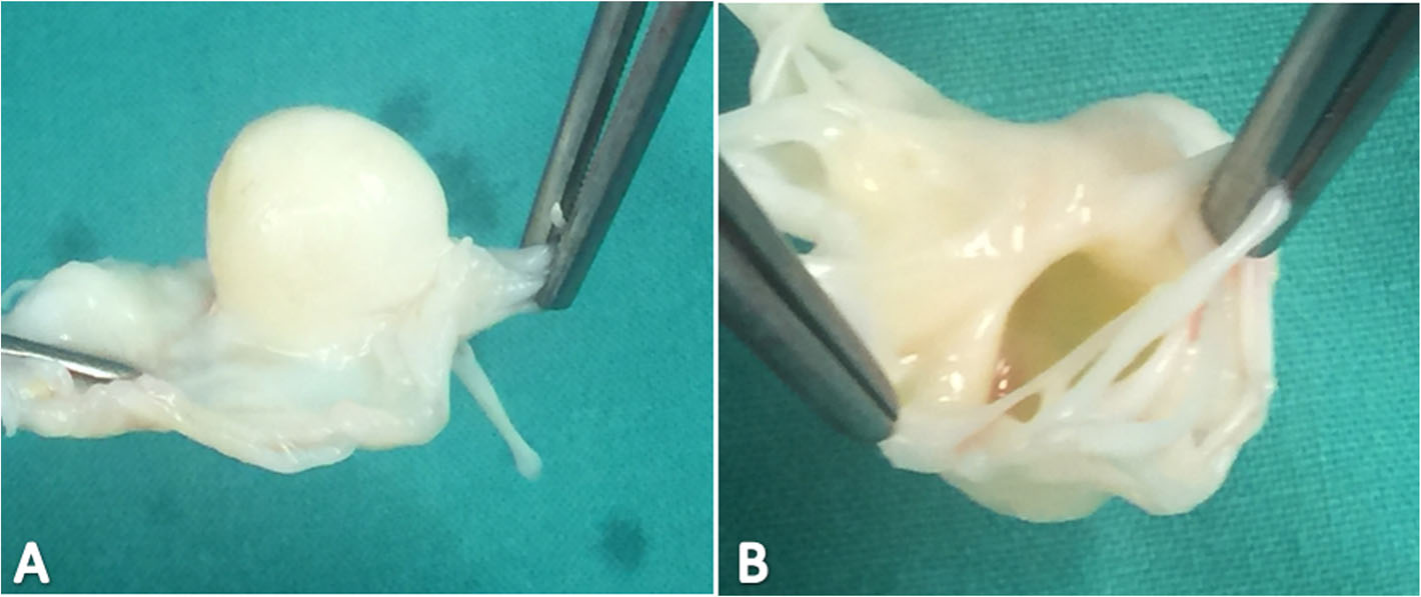
Aneurysm from **a** atrial aspect and **b** ventricular aspect

Subsequently, an elective surgery was scheduled for mitral valve repair +/− replacement. Median sternotomy approach was taken. Cardiac arrest was achieved with antegrade cardioplegia, under cardiopulmonary bypass and the usual state of moderate hypothermia was maintained. Mitral valve was approached through the left atrium and the findings of anterior mitral leaflet aneurysm were confirmed. The severely thickened valve was grossly evaluated again and assessed for repair but it was not possible due to severely distorted leaflets, hence most of the anterior leaflet and part of the posterior leaflet had to be excised (Fig. 3), and replaced with a Hancock II 29 mm valve. On histopathological examination, the valve tissue revealed nonspecific degenerative changes microscopically, with no significant inflammation, atypia or malignancy. Post-operative course of the patient was uneventful; as of this writing, the patient has remained in good health, with regular follow-ups and echocardiography.

## Discussion and conclusions

The incidence of MVA has been reported to be only 0.2–0.29% previously, in patients undergoing TEE [3], with posterior leaflet being much less commonly involved than the anterior leaflet [4]. It is a common sequelae of infective endocarditis (IE) [5]. However, the underlying mechanism for its development is not clearly known. Weakening of the mitral leaflet may be induced by endocarditis, rheumatic disease and other connective tissue diseases like mitral valve prolapse, osteogenesis imperfecta, Marfan syndrome and pseudoxanthoma elasticum [6], causing protrusion of the leaflet in to the left atrium under increased pressure from the left ventricle. In the absence of any other cardiac or systemic abnormality, degenerative changes that result due to aging might be the underlying mechanism leading to leaflet failure and mitral valve aneurysm.

Clinical findings of MVA are similar to those found in mitral regurgitation and there are no signs or symptoms specific to an aneurysm. Perforation is the most threatened complication of MVA, and it results in severe regurgitation. Microscopically, an isolated valve aneurysm is usually found, which may be ruptured, nevertheless, multiple aneurysms have also been reported [7]. Even though theoretically larger aneurysms are more likely to rupture than smaller ones, perforation has been proved to bear no relation to aneurysm size [8].

For appropriate treatment, MVA must be differentiated from other abnormalities with similar findings, including mitral valve prolapse, flail mitral leaflet, myxoma or myxomatous degeneration of mitral valve, diverticulum of mitral valve, and non-endothelialized cyst of mitral valve [6]. Identification through TTE can be uncertain so TEE, a more sensitive and accurate method, is used to confirm the diagnosis [3]. MVA is seen as a saccular mass bulging towards the left atrium, and echocardiography can show connection between the aneurysm and left ventricle [3]. Here, pre-operative TEE played a significant role in diagnosing MVA and severe MR. Due to the ability of three dimensional-TEE to demonstrate a first person’s perspective from the left atrial view, it is a more reliable technique as compared to conventional two dimensional-TEE [9]. Recently, real time three dimensional TEE has shown to provide spatial configuration of cardiac structures and their anomalies in real time [10]. Additionally, pathological investigation showed no features of infective endocarditis or myxomatous changes.

An MVA might be complicated by a rupture, thromboembolism or infection (endocarditis), therefore prompt treatment of valvular aneurysms is necessary. A conservative approach with serial follow-up is suggested for uncomplicated MVA by some authors [3, 5, 6, 8], however, in case of a ruptured aneurysm or a large unruptured aneurysm with severe regurgitation as in the present case, surgical repair/replacement of the mitral valve is the preferred choice. Repair of the valve is not always possible owing to the large area occupied by the aneurysm which might compromise valvular function [11], so replacement is favorable in those cases.

To sum up, MVA is an unusual entity, mimicking mitral valve prolapse or regurgitation clinically, and may occur as an isolated pathology. TEE is an excellent technique to confirm the diagnosis of an aneurysm, and depending upon the severity of the defect, valve replacement is the most suitable treatment modality.

## Data Availability

The patient’s file is under the possession of Aga Khan University Hospital. The datasets used and/or analyzed during the current study are available from the authors on reasonable request.

## Abbreviations

IE: Infective Endocarditis
MR: Mitral Regurgitation
MVA: Mitral Valve Aneurysm
TEE: Trans-Esophageal Echocardiogram
TTE: Trans-Thoracic Echocardiogram
